# For the children? A mixed methods analysis of World Bank structural adjustment loans, health projects, and infant mortality in Latin America

**DOI:** 10.1186/s12992-020-00649-1

**Published:** 2021-01-06

**Authors:** Shiri Noy

**Affiliations:** Department of Anthropology and Sociology, Denison University, 100 West College Street, Knapp Hall 103-D, Granville, OH 43023 USA

**Keywords:** World Bank, International financial institutions, Health policy, Infant mortality, Structural adjustment programs, Latin America

## Abstract

**Background:**

The World Bank wields immense financial and normative power in health in the developing world. During the 1980s and 1990s, in the face of intense criticism of its structural adjustment policies, the World Bank purportedly turned its attention to “pro-growth and pro-poor” policies and new lending instruments. One focus has been an investment in maternal and infant health. My analysis uses a mixed methods approach to examine the relationship between traditional structural adjustment and health loans and projects and infant mortality in Latin America and the Caribbean from 2000 to 2015.

**Results:**

My answer to whether the World Bank’s projects in Latin America worked “for the children” is: somewhat. The results are heartening in that quantitatively, health projects are associated with lower infant mortality rates, net of controls, whereas traditional structural adjustment loans do not appear to be negatively associated with infant mortality, though examined across a short time horizon. Qualitative data suggest that infants, children, and mothers are considered in World Bank loans and projects in the context of an economic logic: focusing on productivity, economic growth, and human capital, rather than human rights.

**Conclusion:**

Taken together, my results suggest that the World Bank appears to, at least partially, have amended its approach and its recent work in the region is associated with reductions in infant mortality. However, the World Bank’s economistic approach risks compartmentalizing healthcare and reducing people to their economic potential. As such, there remains work to do, in Latin America and beyond, if health interventions are to be effective at sustainably and holistically protecting vulnerable groups.

The effect of international financial institutions on health policies and population health outcomes has received intense attention in recent years [10, 16, 25, 40, 41]. Scholars have argued that structural adjustment programs, promoting neoliberal, pro-market reforms, have historically had adverse effects on health outcomes. Other research finds that the effects on national policies and outcomes beyond population health, such as public health expenditures, may be variable, especially across world regions but also across international financial institutions (IFIs, e.g. the International Monetary Fund as compared with the World Bank). The World Bank, historically the largest single external funder of global health [46], has deepened and broadened its engagement with health in developing countries in recent decades. Latin America, in particular, has often served as “laboratory” of sorts for World Bank and government partnerships [33] and the World Bank remains the largest single external funder of global health in Latin America, though this is not true across all world regions [40]. However, we know little about the relationship between World Bank loans and projects and health outcomes in the region, despite information about historical deleterious effects of structural adjustment programs on health in sub-Saharan Africa.

This article begins to fill this gap by conducting a mixed methods analysis of World Bank projects and infant mortality and framing of infant and maternal health in Latin America. Using regression models that control for selection bias, I find that World Bank structural adjustment programs are not associated with higher rates of infant mortality, as scholars have found they are in sub-Saharan Africa, and in contrast to findings that IMF structural adjustment programs are positively related to infant mortality globally. To contextualize this finding, I examine the framing of health and maternal and infant health in select loan project documents and find variability: while the World Bank appears sensitive to population health risks the logic is still economistic: health is framed as a desirable investment because of its effect on the labor pool and economic growth. These findings suggest that we need to revisit our information about the World Bank’s work in health, and provides insight about how programs that favor economic growth and stability may be pursued without corresponding declines in population health.

## Background

### The World Bank and health

While the proliferation of new and large global health funding agencies, perhaps foremost the Bill and Melinda Gates foundation, has changed the funding landscape of global health, international organizations remain important actors in the field [36]. The World Bank remains a uniquely powerful agency for several reasons. First, in some regions, such as Latin America, it remains the single largest external funder of health [40]. Second, while its share of international health funding may be diminishing among new funders, the World Bank still wields a powerful and singular voice among its global health peers. Its historical domination of the global health funding field (since it began direct lending for health in 1980) means that it has built up a reputation over many years and is still regarded as an important authority in this field. Third, it conducts important data gathering and disseminating work: many development scholars use statistics compiled by the World Bank—the World Development Indicators, and its technical and other projects may be the only data gathered in developing countries about particular disease and health challenges in some places. Fourth, its funding mechanisms of providing loans with attached conditions, together with its status as a funder of last resort to national governments means it can suggest (and sometimes impose) measures on countries in return for funding [9].

The World Bank and other IFIs have been criticized for taking a neoliberal approach: one that prizes market mechanisms and efficiency over state involvement and equity concerns [32]. Despite its stated goal of “pro-poor and pro-growth” efforts the Bank has been heavily censured for its development work. Among the more salient concerns are allegations that it prioritizes the concerns of the U.S., Europe, and other developed countries over those of developing, low- and middle-income countries [1]. Related, it has been charged with a lack of sensitivity to developing country needs and priorities, and criticized for not being attentive to issues of gender, sexuality, and other important dimensions of inequality and oppression. Finally, the World Bank has been accused of pursuing economic growth at the expense of people in instituting draconian structural adjustment programs leading to poverty and unemployment, though the Bank counters there are short-term challenges in the pursuit of long-term growth and has pursued new lending instruments [20, 23].

Existing analyses of structural adjustment programs often treat the World Bank and IMF interchangeably since they both have utilized structural adjustment policies. However, this may be a mistake because of their different purposes and agendas, as well as foci [1]. Scholars are increasingly recognizing that the World Bank’s work is variable across regions and sectors, in addition to being different from that of the IMF. There is reason to believe that the effects of its loans and projects varies not only across countries, world regions, over time, but also across domains [40]. That is, pushes to privatize state-led industries may have different effects than implementing education and health projects, which in turn may be different from each other. In health, recent research suggests that the World Bank has been increasingly pushing what many consider progressive ideas, such as supporting universal healthcare and concerning itself with equity in health [15] as well as supporting important domestic poverty alleviation programs more specifically in Latin America, such as the *Progresa-Oportunidades* program in Mexico [38]. Recent evidence from the developing world suggests that World Bank funding is associated with better health outcomes, such as access to water and infant mortality, further supporting indications of movement in the right direction [13, 19].

### Structural adjustment and infant mortality: trends and exceptions

Despite these strides on the part of the World Bank, there is considerable evidence that structural adjustment programs may have had adverse effects on infant mortality. In particular, previous research has found that in sub-Saharan Africa the presence of World Bank, African Development Bank, and IMF projects has adverse effects on maternal and infant mortality [10, 47], demonstrative of its organized hypocrisy [56]. A recent, broader review found that structural adjustment programs limit access to quality healthcare, and adversely impact maternal and infant health [54]. This important research is often focused on the IMF and largely based on insights from Africa, and particularly sub-Saharan Africa (cf. [11, 53, 65]). However, it is unclear whether and how these trends may extend to other world regions, such as Latin America.

There is some reason to believe that patterns in sub-Saharan countries and other developing regions may not track identically to Latin America. First, there is evidence that while structural adjustment programs and IFI loans have reduced public commitments to health in the form of government health expenditures in the developing world [26], these effects have not been as evident in Latin America, even across different time periods [22, 24, 39, 40]. Second, much of the literature has focused on the IMF [14, 50], rather than the World Bank, whose structural adjustment programs may have different effects given the different mandates of these institutions and the World Bank’s explicit focus on health. Third, the World Bank operates via regional offices suggesting variable approaches across global regions and even within regions, country offices may have different priorities. Therefore, the Bank’s work is different across countries, and often contingent on government needs, capacity, and agendas with Latin American countries sometimes challenging structural adjustment efforts [35, 41]. Fourth, the World Bank has changed its approach over time, and has amended its tactics (though the extent is debatable), never abandoning its focus on economic growth but increasingly focused on being “pro-poor” in addition to being “pro-growth” and concerning itself with social inequities along gender lines, for example [3]. It has also updated its lending instruments, including adopting development policy financing that combines and merges several lending instruments including structural adjustment which have been shown to be related to positive social sector outcomes [5].

Additionally, World Bank interventions and recommendations could also positively influence health outcomes, for example, fiscal decentralization which is a popular World Bank policy recommendation is associated with better infant health outcomes in Colombia (though these effects are stronger in wealthier municipalities, [49]). Finally, Latin America has been a political and social laboratory of sorts, in terms of democratization, economic development, and also in some ways in terms of the World Bank’s work in the region [21, 33, 57]. The question of whether regional patterns in Latin America mirror those of other developing countries and whether there have been changes in recent years requires empirical exploration and has important implications for our understanding of the World Bank’s work in global health and health outcomes, such as infant mortality.

### Infant mortality, health, and development

Infant mortality has long been of interest to both health researchers and development scholars. It is an important indicator not only of population health but of broader national conditions such as national health and economic systems and of social development. Infant mortality also captures governments’ commitments to vulnerable segments of the population who lack, for example, political agency via voting, as is the case of infants and children. In particular, infant mortality is an indicator of prenatal and postnatal care, but is also suggestive of roadways and transportation, access to physicians and other health professionals, vaccination, poverty and nutrition, and other important public goods. As such, examining infant mortality conveys important information about the state of health in particular countries as well as about levels of development. In many ways, infant mortality and other health indicators may more accurately capture development than economic indicators like GDP (which albeit related) do not address questions of vulnerability, inequality nor questions of how growth and wealth may translate to population health and social well-being.

Infant mortality has increasingly been used to capture development in a single, summary metric, and has become particularly popular among international agencies because of its encompassing nature. Indeed, the Millennium Development Goals (MDGs) and Sustainable Development Goals (SDGs) both focus on maternal and infant mortality as an important goal of broader national development. The SDGs, and their predecessor the MDGs authored by the UN, may be the most well-known development indicators globally, and have fueled countless publications, conferences, and country-level competitiveness in reaching benchmarks. In particular, the SDGs set a goal to reduce infant mortality and maternal mortality to certain benchmarks by 2030 [55]. However, there is a danger that this focus on maternal and infant mortality may be caught up in other neoliberal discourses, and that investments in mothers and children are parsed away from the broader contextual features of health and other economic systems. Additionally, there is a concern that this focus on mothers and children follows a utilitarian logic, where the primary concern is with expanding future labor pools, following a human capital rather than rights-based approach [6, 10, 41]. That is, if a focus on mothers and children is divorced from broader conversations about inequality, gender, and social protection it risks first, alienating broader support (for example, from upper and middle-class segments of society) putting it at danger of becoming a temporary measure, and second, failing to address systemic issues by providing “band aid” solutions to the underlying causes of infant mortality [41].

While this focus on gender in international aid and development organizations is not new, dating back to the 1960s and 1970s [27], the focus on mothers and children and in particular their health, has intensified given the focus on these metrics in the MDGs and SDGs [34]. While authored by the UN they have been enthusiastically embraced by the World Bank, a member if UN system. In these ways, the World Bank contributes to and delineates global health priorities globally and especially in developing countries, which also inform its lending and investment practices and may be associated with differential health outcomes.

## Data and methods

In order to examine the relationship between World Bank structural adjustment and health projects and infant mortality in Latin America I utilize a mixed methods approach drawing on both qualitative and quantitative data. In particular, I use a sequential mixed methods design [12] where the quantitative data provide an overview of regional trends while qualitative data gleaned from World Bank project and loan documents serve to help illustrate and provide additional insight about the patterns of the quantitative data.

First, I draw on country-level quantitative data for countries in the region over time between 2000 and 2015. Second, to contextualize regression results I utilize policy documents to examine the framing and discussion of infant mortality in World Bank projects in the region during the same time period.

### Quantitative data and analytic strategy: infant mortality, structural adjustment, and health loans

Regional quantitative data is drawn from the World Development Indicators for 18 Latin American and Caribbean^1^ countries over 16 years: 2000 to 2015 [63]. The data is unbalanced panel data, meaning that there is missing information for some country-years owing to unavailable data on at least one of the variables used in the analysis. Infant mortality, the dependent variable, is captured as the rate of infant mortality for children under a year of age per 1000 live births from the World Development Indicators [63]. My primary independent variable of interest is the presence in a given country-year of a World Bank structural adjustment loan (SALs). I also examine the presence of the World Bank health projects. These variables were coded from the World Bank projects database, for a SAL by whether it as identified as a structural adjustment loan (including programmatic structural adjustment loans). Health projects were coded according to the type of loan (these include adaptable program loans, emergency recovery loans, health sector adjustment loans, investment project financing, learning and innovation loans, sector investment and maintenance loans, and specific investment loans) as well as primary sector and years were coded based on start and end years in the dataset, provided as board approval and closing dates [64].^2^ The health projects are heterogeneous, as the examples below demonstrate, including projects that focus on insurance and financing, others that target particular diseases, such as HIV/AIDS while some focus on maternal health. Overall, structural adjustment loans lasted an average of 20 months and averaged $242 million in total amount, while health projects lasted an average of 70 months and with an average of $114 million in total amount. Finally, I employ a two-step process to calculate and include an Inverse Mills ratio to capture selection bias, detailed below.

I also include a number of controls that capture a variety of economic, political, and social conditions. In particular, I measure public health expenditure per capita to allow for comparability of health spending across heterogeneous economies and populations in the region. I expect that countries with higher national health investments will have lower rates of infant mortality [17, 37]. Similarly, because many of the causes of higher infant mortality are associated with infectious diseases including dysentery, I control for sanitation services. I also control for GDP per capita, to capture country-level economic development, which I expect to be negatively associated with infant mortality. I also utilize a measure for the level of democracy as different types of political regimes have been associated with infant mortality and access to healthcare [18], though the relationship is complex [7]. As indicators of globalization I include net Foreign Direct Investment (FDI) and trade volume, the sum of imports and exports, as a percent of GDP. I expect that these measures may be positively related to infant mortality if trade and competition over foreign direct investment drive “race to the bottom” globalization whereby countries compete for foreign investment by reducing commitments to environmental, labor, and social protections [45]. On the other hand, trade and foreign direct investment may bring new knowledge flows, technologies, and strategies and reduce the prices of medical supplies ([4]; for a recent review of the relationship between trade and health see [28]). Finally, I include time as year in a linear measure.

To analyze the data, I estimate cross-section time-series models to examine how the variables detailed above are related to infant mortality rates in Latin American and Caribbean countries. In this regression, the outcome, the infant mortality rate in country *i* during year *t* is modelled as a function of the presence of a structural adjustment loan (*SAL*_*it*_), World Bank health projects and controls included in the model (*C*_*it*_), as well as a linear term for year (*t*), the constant (*α*_0_) and an error term (*ε*_*it*_).

1 $$ {IMR}_{it}={\alpha}_0+{\beta}_2{SAL}_{it}+{\beta}_3{C}_{it}+t+{\varepsilon}_{it} $$

However, the presence of a World Bank structural adjustment loan in a given country during any particular year is not random: countries must propose projects that the World Bank agrees to fund (often following negotiations). This poses an estimation problem, as the factors that influence whether a country receives a structural adjustment loan may be associated with infant mortality rates. Some of these variables are already accounted for in the model: for example, GDP per capita captures countries’ wealth which may associated with both receiving structural adjustment loans and infant mortality. Other variables are harder to operationalize. For example, countries that are motivated to seek structural adjustment loans may also be more likely to address infant mortality as they seek to implement system-wide political reforms. On the other hand, countries seeking structural adjustment loans may be particularly vulnerable and less concerned with social development as compared with economic stability. Indeed, previous analyses have found evidence for selection effects, though these have sometimes focused on the IMF rather than the World Bank and on regions other than Latin America (cf. [42, 51]).

In order to address this issue, where those countries that “select” into World Bank loans may be different than those that do not in ways that may also affect the outcome variable of infant mortality, I draw on a variant of Heckman’s (1979) two-step approach which may be particularly useful when collinearity is a concern [43] and which first utilizes a probit regression model where the outcome is selecting into World Bank structural adjustment loan. This regression equation can be modelled as:

2 $$ {SAL}_{it}=\gamma {Z}_{it}+{\upeta}_{it} $$

where *Z* are the measured covariates which may predict the presence of a World Bank loan in a given county *i* in year *t* and a stochastic component, η_*it*._

The covariates included in this first step, that is, *Z*_*it*_ in eq. (2) are: GDP per capita, democracy score, FDI, trade, external debt stocks, and whether the country had a World Bank structural adjustment loan in the previous year. These variables were selected following existing literature on structural adjustment and substantive considerations. That is, we might expect poorer countries as well as those with higher amounts of debt and also countries that had an active SAL in the previous year to have a SAL in a particular country-year while trade might reduce the probability of having a SAL [16, 40, 57]. A recent review focused on IMF conditionality, using the empirical case of education, suggests that this type of Heckman variant approach is an efficient approach to the issue of selection bias encountered when examining international financial institution projects [52].

If there is selection bias, the stochastic component (η_*it*_) in this second eq. (2) will be correlated with the error term (*ε*_*it*_) in the regression equation predicting the IMR (1). In order to test whether this is the case, I calculate the “inverse mills-ratio” (3) using the following equation where φ is the standard normal density function, Φ the standard normal cumulative distribution function, and $\hat{\upgamma}$ is an estimated value taken from eq. (2).

3 $$ {\hat{\lambda}}_{it=}\ \frac{\upvarphi \left({\mathrm{Z}}_{it}\hat{\upgamma}\right)}{\Phi \left({\mathrm{Z}}_{it}\hat{\hat{\upgamma}}\right)} $$

I then add the inverse-Mills ratio to eq. (1). Once added to the full model as an additional control the coefficient indicates whether there is selection bias. If it is not statistically significant we do not find an association between selection into a World Bank SAL and the IMR. If the coefficient is positive and statistically significant this suggests that unobserved variables that affect countries taking out World Bank structural adjustment loans are also associated with higher infant mortality rates, and if the coefficient is negative and statistically significant the opposite is the case. Table 1 provides descriptive statistics for the variables included in the analysis.

**Table 1:** Descriptive Statistics for Variables Included in the Analysis

	Mean	Std. Dev.	Min	Max
Dependent Variable				
Infant Mortality Rate (aged under one years old, per 1000 live births)	24.01	13.06	7.8	85.6
Independent Variables				
World Bank Structural Adjustment Loan (SAL)	0.22	0.41	0	1
Inverse Mills Ratio	2.42	1.48	0.02	6.37
World Bank Health Project	0.71	0.45	0	1
Controls				
Public Health Spending per capita (logged, current US$)	4.50	1.12	0.86	6.92
% of the Population Using Basic Sanitation Services	74.40	17.26	16.75	97.27
GDP per capita, PPP (logged, current international $)	8.88	0.61	7.22	9.91
Democracy Score	7.27	2.32	-3	10
Net FDI (hundreds of millions, current US$)	−4.67	11.78	−90.49	9.42
Trade (total volume of exports plus imports, % of GDP)	69.12	32.00	21.85	206.77
Year	2007.51	4.57	2000	2015

Source: World Development Indicators, Polity IV, World Bank Projects Database (author coded)

Notes: Sample size is 279 country-years in 18 countries over 16 years (data is unbalanced panel data)

### Qualitative data and analytic strategy: World Bank loan and project documents

Qualitative data are drawn from documents associated with 57 structural adjustment loans and 72 health investment projects and loans provided by the World Bank to Latin American countries between 2000 and 2015 and included in the quantitative analysis. I draw from documents including loan agreements as well as implementation and completion reports among other project documents. Not all loans had all associated documents published though the analysis presented here is intended to be illustrative rather than exhaustive. I examined documents associated with these projects primarily to provide context for the cross-section time-series regression results, focusing on how maternal and infant health is discussed: focusing on framing, outcomes, and priorities. In this way, the qualitative data serves to illustrate and clarify the quantitative results, particularly the framing of the relationship between health, maternal and infant mortality, and issues of poverty, economic growth, and labor as these are perennial concerns for the World Bank. While regression models are well suited to provide information about broad trends and correlates of infant mortality in the region, including World Bank projects, they do not provide information about the discourses and framings surrounding these projects. Loan and project documents provide illustrative information about the World Bank’s stated motivations, justifications, and framings of infant mortality and health itself.

In this way, the qualitative data supplements and contextualizes the quantitative data in a sequential mixed methods design. This data allows at once a broader view of how health is conceptualized by the World Bank in select projects in Latin America during this period as well as how maternal and infant health fit into both health investment and structural adjustment lending. Importantly, these documents provide a “public transcript” of these projects, rather than a behind the scenes view of how decisions were made and negotiations, and in this way are perhaps sanitized to make the World Bank appear in a more positive light. Therefore, I use them to document the World Bank’s *stated* aims, rather than arguing that they represent its effects. However, these documents are valuable as they reflect the publicly available and agreed upon—between national governments and the World Bank—record of how the World Bank thinks about and conceptualizes its work in health and its focus on infant mortality.

## Results

### Structural adjustment, health projects, and infant mortality

Table 2 contains results from the cross-section time-series regression models. Models 1–3 provide random effects, cross-section time-series bivariate analyses of the effects of World Bank SAL and health projects on infant mortality.^3^ Model 1 suggests that at the bivariate level World Bank SALs are not statistically significantly associated with a higher nor lower infant mortality in Latin America. Results in Model 2 indicate that health projects are associated with lower infant mortality, where the presence of a health project is associated with a lower IMR of approximately 1.7. Model 3 indicates that when these two types of lending instruments are introduced jointly the effects remain unchanged: SALs do not appear to be associated with differential rates of infant mortality while health projects are associated with lower rates of infant mortality.

**Table 2:** Regression of Infant Mortality Rate on World Bank SAL and Health Projects, and Select Controls

	Model 1	Model 2	Model 3	Model 4	Model 5	Model 6	Model 7	Model 8
World Bank Structural Adjustment Loan (SAL)	0.17 (0.47)		0.02 (0.46)	0.02 (0.46)		−0.22 (0.44)	0.46 (0.52)	0.17 (0.50)
World Bank Health Project Loan		−1.66*** (0.41)	−1.66*** (0.41)		−1.74*** (0.37)	−1.75*** (0.37)		−1.71*** (0.37)
Inverse Mills Ratio							0.59+ (0.33)	0.52 (0.32)
Public Health Spending per capita (logged, current US$)				2.27*** (0.65)	1.83** (0.62)	1.79** (0.64)	2.21*** (0.65)	1.75** (0.63)
% of the Population Using Basic Sanitation Services				−0.55*** (0.07)	−0.56*** (0.06)	−0.56*** (0.07)	− 0.56*** (0.07)	−0.56*** (0.07)
GDP per capita, PPP (logged, current international $)				−6.22** (2.05)	−5.96** (1.99)	−6.06** (2.01)	−5.78** (2.09)	−5.70** (2.05)
Democracy Score				−0.25+ (0.14)	−0.25+ (0.13)	− 0.23+ (0.14)	−0.15 (0.15)	− 0.14 (0.14)
Net FDI (hundreds of millions, current US$)				0.01 (0.02)	0.01 (0.02)	0.01 (0.02)	0.04 (0.03)	0.03 (0.02)
Trade (% of GDP)				−0.02+ (0.01)	−0.02 (0.01)	− 0.02 (0.01)	−0.03* (0.01)	− 0.03* (0.01)
Year	−0.79*** (0.04)	− 0.84*** (0.03)	−0.84*** (0.04)	− 0.34*** (0.07)	−0.35*** (0.07)	− 0.35*** (0.07)	−0.47*** (0.10)	`-0.46*** (0.10)

Source: World Development Indicators, Polity IV, World Bank Projects Database (author coded)

Notes: Random effects models. Standard errors in parentheses; +*p* < 0.10; * *p* < 0.05; ** *p* < 0.01; *** *p* < 0.001 (two-tailed tests)

Sample size is 279 country-years in 18 countries over 16 years (data is unbalanced panel data)

In Models 4–6 I introduce control variables. Multivariate results once again suggest that either alone (Model 4) or jointly with health bank projects (Model 6) SALs are not associated with improved or diminished infant mortality in Latin America, net of controls. Health projects remain associated with lower infant mortality rates (Models 5 and 6), all else equal. In terms of controls, Models 4–6 suggest that access to basic sanitation services as well as higher GDP per capita and democracy are associated with lower infant mortality rates, as expected. Net of controls (Models 7 and 8) trade is associated with lower infant mortality rates which is consistent with some recent research that finds a positive relationship between trade liberalization and reductions in infant mortality, particularly in Latin America [2]. While public health expenditure appears to be associated with higher infant mortality rates, this effect is only apparent net of controls, and particularly GDP per capita, and at the bivariate level there is no statistically significant association.

However, as discussed above and as existing research on IMF and World Bank structural adjustment programs suggests, there may be selection effects. That is, countries that are more likely to take out SAL from the World Bank may also have other unobserved characteristics, which make taking out a SAL more likely, but which may also be associated with higher or lower rates of infant mortality. For example, if a government has clear vision for reforming and growing the country’s economy, it may also be more likely to have been politically motivated to address major health challenges. Conversely, if a government is willing to implement strict and difficult reforms such as structural adjustment it may also be less concerned with health outcomes (perhaps especially in the short term) and may be under pressure to control inflation, etc. rendering health and other social policy concerns secondary.

### Testing for selection effects

In Model 7 I employ Heckman’s two-step procedure to test for such selection. While the time period is short, the results do not suggest selection effects. Upon the inclusion of health projects in Model 8 the inverse mills ratio is marginally statistically significant, but the coefficient for a SAL remains statistically insignificant.

These findings stand in contrast to some existing research about the World Bank’s work in other regions and in previous time periods: namely findings that suggest the World Bank’s SALs are associated with higher infant mortality rates in sub-Saharan Africa [47] and about the effect of structural adjustment by the IMF in other world regions [25]. However, they are consistent with previous quantitative research which has found that despite high IFI activity in the region, public health expenditures in Latin America have not decreased from the 1980s onward and also dovetails with qualitative research outlining the World Bank’s varied approach to health in Latin America and diverse effects of structural adjustment and IFI projects on health [22, 24, 31, 33, 40, 41].

My results suggest that while these may not work at cross-purposes per se, traditional structural adjustment loans are not associated with differential rates of infant mortality during this time period whereas health projects are associated with lower infant mortality rates (Model 8). How can we understand these differences? And while the overall relationship suggests World Bank projects are associated with lower rates of infant mortality, how does the World Bank frame this work in health? An examination of select documents associated with loan agreements and projects provides some preliminary insight into how the World Bank frames its work on infant and maternal health.

### Illustrating the World Bank’s framing of health: contextualizing regional models

Quantitative, regional analyses allow an examination of whether and how structural adjustment and health projects may be related to infant mortality. However, they do not give us a sense of how the World Bank conceptualizes and frames investments and projects focused on infant and maternal health. While I find that World Bank health projects are associated with lower rates of infant mortality on average, structural adjustment programs do not appear to be related to infant mortality in either direction in Latin America since 2000. Given the literature on the ill-effects of structural adjustment programs for other regions how can we interpret these findings in Latin America? Existing research suggests that this may be the result of particular traits of the Latin American context, motivating regional analyses on the effects of global institutions. However, this research also suggests that the World Bank’s approach may have changed and that its effects on health are contingent on national institutions. Therefore, structural adjustment programs may be more attentive to social development outcomes since 2000 given the lessons learned from their deleterious effects in the 1980s and 1990s. There is also the possibility, however, that programs vary significantly across countries, whereby structural adjustment may have negative effects in some places, neutral or positive effects elsewhere and this “washes out” in the regional analysis. Qualitative data from loan documents allows contextualization of the regional results, and an illustration of how some of these projects discuss maternal and infant health.

### Structural adjustment loans: efficiency and economic growth

In order to contextualize the regression results, I examine the framing of health and infant mortality in loan documents. I find some evidence that suggests a clear recognition of the possible detrimental effects of structural adjustment by the Bank and national governments. There is also recognition of the need to concurrently support social expenditures with a focus on efficiency on behalf of governments seeking loans, though this is framed using economic rather than rights-based logics. For example, in Guatemala the proposal for a Financial Sector Adjustment Loan in 2002 includes a letter from the national Minster of Finance and President of the Bank of Guatemala which notes “Over the long run, the gradual integration of the large rural indigenous population in the market economy, together with the strengthening of human capital through poverty reduction and access to basic education and health, should raise the productivity of labor and expand the domestic economy …To guide its politics in this respect, the Government has prepared a poverty reduction strategy paper that will complement its economic program” ([58], p. 2). The fact that this explicit attention to poverty reduction amidst structural adjustment features in a World Bank loan provides an indication that the issue of basic services, in terms of health and education, are being given consideration. While the World Bank discusses a “pro-poor and pro-growth” approach, these concerns are often tied to efficiency goals and benchmarks—often the MDGs and SDGs, which may risk compartmentalizing health care [29].

An earlier structural adjustment program in Argentina during the same time period too considered social services and notes that “As with all structural adjustment loans, the SAL-I also aimed generally to support macroeconomic stability” ([60], p. 2). While the implementation and completion report discusses issues of quality and equity in health, this is in tandem with efficiency, and the underlying concern was primarily financial and economic: “Health conditionality under the SAL-I was designed to consolidate ongoing reforms in health insurance supported by three previous IBRD [International Bank for Reconstruction and Development, an arm of the World Bank] operations (Health Insurance Reform Loans 4002-AR, 4003-AR and 4004-AR) amounting to a total of US$375 million. The objective of these operations was to improve the efficiency and long-term financial solvency of the major health insurers (known as *Obras Sociales, OS*)” ([60], p. 4).

As another illustration, a project in Colombia aimed to, among other things, reduce costs and increase efficiency in the health sector focusing on hospitals, where the completion report notes that the objective “of the operation was to support the government’s commitment to improve Colombia’s fiscal accounts and implement structural fiscal reforms, an essential first step toward reaching full fiscal sustainability, sustaining high economic growth and achieving poverty reduction” ([59], p. 2) including by focusing on efficiency and productivity in hospitals. The connection between poverty reduction and high economic growth together with efficiency and productivity measures are bundled as stated goals in this project, though not elaborated at length.

This structural adjustment project also demonstrates how SALs may be neutrally associated with health equity and other outcomes, in supporting reforms that may simultaneously affect infant mortality negatively and positively. For example, one SAL demonstrates support for a fund that redistributed resources among unequal health insurance funds (in Argentina these are organized along occupational lines: the *Obras Sociales*). In the same SAL however, another condition mandates free choice among health insurance funds which may serve to increase inequality as individuals and families with more resources may opt into bigger, better financed funds. Similarly, the SAL seeks to eliminate double-coverage and enhance targeting efforts by developing a national registry of beneficiaries of federal assistance programs, thereby potentially increasing administrative costs and barriers to care.

### Health projects and investment loans: human capital and cost control

While structural adjustment loans do not target health per se, and appear to have a neutral relationship with infant mortality in Latin America albeit over a comparatively short time horizon, as demonstrated in the regression models, health projects appear to be associated with reductions of infant mortality, net of controls. However, the framing in health investment projects suggests that addressing infant mortality is still tied to discourses surrounding economic growth but also poverty. As an example, the Project Appraisal Document for a project on Maternal and Infant mortality in Guatemala provides a rationale for focusing on maternal and infant investment as the risk and reality of “irreversible losses of human capital formation, affecting current and future generations and undermining economic growth” ([61], p. 1). Therefore, infant mortality is still framed as an important economic, rather than moral, consideration in this health project.

A more recent health investment loan to Argentina focuses on non-communicable diseases (NCDs), and in fact notes that perhaps the World Bank has focused its work too narrowly on maternal and infant health at the expense of the elderly and other vulnerable populations, specifically the poor. This suggests a possible shift away from the focus on women and children by the World Bank, given the increased prevalence of NCDs. However, even projects not explicitly focused on maternal and infant health may have positive spillover effects. For example, in motivating the project the World Bank notes: “There is a strong association between poverty, nutrition, and NCDs. With increasing urbanization the cost of fresh foods, especially fruits, vegetables and meat, has increased; while processed foods have become much cheaper” ([62], p. 3). Interventions include developing clinical information systems. Therefore, while targeted at NCDs such projects may have important impacts for health outcomes across the spectrum, including infant and maternal health. Importantly, this project too frames these issues in economic terms: “If left untreated or uncontrolled, they may result in costly hospitalizations, thereby generating an important negative economic impact to households, the health system, and the economy” ([62], p. 2). While this framing is sensitive to impacts on households the emphasis here too is primarily economic.

## Discussion

Overall, the results presented in this article suggest several important insights: first, my analysis does not find strong evidence of a selection effect into World Bank structural adjustment programs in Latin America between 2000 and 2015. However, there are several important caveats: the time period under examination is short, and testing for selection is not the primary goal of the analysis, which seeks rather to mobilize quantitative and qualitative data to examine whether and how World Bank loans across types are associated with infant mortality; second, there is no relationship, on average, between structural adjustment loans and infant mortality in the region, for better or worse; third, World Bank health projects, as compared with SALs, are associated with lower rates of infant mortality in Latin America during this time period, net of controls.

These results add to the stock of existing information about structural adjustment and health outcomes in other regions and time periods which often find negative effects of IFIs on health outcomes. This may owe to regional differences but also the World Bank’s changing approach to health and development, which has also included changes to lending instruments. However, it is also consistent with existing literature that has noted regional dynamics of health systems in Latin America and changes over time. Altogether, the quantitative results speak to the importance of contextual, regional analyses for better understanding the effects of international organizations on health outcomes. The qualitative analysis presented in this article sheds light on the goals and framing of World Bank structural adjustment and health projects as related to health in general, and infant and maternal health in particular, which still follows an economistic logic.

My qualitative analysis suggests that while both health investment projects and some structural adjustment projects are argued to be “for the children” they frame this goal differently. Structural adjustment programs appear to seek to promote efficiency, economic growth and stability, though they sometimes demonstrate a concern for poverty alleviation and buffering against the possible attendant ill effects of adjustment on population health, along with other indicators. Equity is to be pursued via efficiency, following economic objectives. In health projects the logic is also economic and focused on productivity and economic stability, though the focus is longer term and concerned with poverty, and there may be spillover possibilities. That is, even health projects that focus on non-communicable diseases seek to improve nutrition and other interventions which may have wider impacts, including on infant mortality. Another question however, is that of the sustainability of projects with positive interventions which may be limited by the finite time-frame of external funding.

This study suggests important directions for future research: first, while the time period under consideration here provides information on more recent years in Latin America, extending existing research, new instruments employed World Bank require more detailed attention to update our understandings of their effects on population health. Further, the time horizon examined is comparatively short and the paper does not examine conditions associated with loans, for example, which may provide additional insight on how conditionality may influence infant mortality. Second, the estimation of selection effects is an ongoing area of inquiry, particularly as instruments are difficult to find for these analyses [30] and while these results should not be taken as the final or definitive word on selection into structural adjustment loans they examine selection using a popular strategy [48, 52] and are consistent with recent research on the effects of IFIs on health outcomes [13, 19]. Third, the quantitative analysis in this article is not intended to make causal claims and while the qualitative analysis in this paper provides insight into how World Bank frames infant mortality across lending instruments it is does not provide detailed information on pathways of influence—recent research focused on the IMF suggests important possibilities in terms of women’s labor force participation and public polices [25] and future research should focus on possible mechanisms as well as additional outcomes, such as other measures of population health including specific disease-related mortality. Additionally, Latin American governments and institutions, in terms of their autonomy, capacity, and other characteristics may be important contributors to the relationship between World Bank activity and infant mortality described in this paper [40], and while supplementary models with country fixed effects control for the unobserved country characteristics suggest similar conclusions the Bank-government relationship merits additional attention. Fourth, the qualitative analysis is illustrative, and future work can more systematically and comprehensively compare discourses surrounding mothers, infants, and health across types of projects both in Latin America and beyond to ascertain whether and how the findings presented here extend more exhaustively within the region and beyond.

## Conclusion

Infant mortality is an important indicator of population health and development however, the increased focus on infant mortality must also be considered critically: as social scientists have warned, there is danger that the measure becomes the goal rather than being a measurement or indicator of the goal [8]. In those cases, the measure may cease to be a good indicator because it has been co-opted as an outcome and therefore no longer serves as a useful gauge of progress towards the broader goal. In the case of infant mortality, the IMR may serve as a targeted metric, which while an important goal, diminishes its utility as a broader indicator of health equity and a focus on vulnerable groups, population health, and investment in health. However, while IMR has been criticized as a measure it is not clear that other indicators are better suited as summary measures of population health, where other indicators are often more complex and require additional funds to calculate, risking distributing funds towards measurement rather than health interventions [44].

Taken together, my analysis suggests that the question of whether World Bank structural adjustment and health projects in Latin America have been “for the children” is: somewhat. In terms of framing, many of these projects do not ignore population health and adverse outcomes during this time period. But, inasmuch as infants, children, and mothers are a focus of World Bank projects and loans this follows an economic logic: children are often viewed as future labor, a question of human capital, productivity, and economic growth, rather than rights. This risks compartmentalizing healthcare and reducing people to their economic potential. However, the results are heartening in that health projects are associated with lower infant mortality rates in Latin America during this time period. Taken together, this suggests that the World Bank may have amended its approach though there remains considerable work to do if it seeks reduce infant mortality and other adverse health outcomes, both in Latin America and beyond.

## Data Availability

Quantitative data and script file to generate the results presented in the paper will be made available for replication purposes.

## Footnotes

1 The countries included in the analysis are: Argentina, 2000–2015; Bolivia, 2000–2015; Brazil, 2000–2015; Colombia, 2000–2015; Costa Rica, 2000–2015; Dominican Republic, 2000–2015; Ecuador, 2000–2015; El Salvador, 2000–2015; Guatemala, 2000–2015; Guyana, 2000–2015; Haiti, 2000–2015; Honduras, 2000–2015; Jamaica, 2000–2015; Mexico, 2003–2015; Nicaragua, 2000–2015; Paraguay, 2000–2015; Peru, 2000–2015; Venezuela, 2005–2011.

2 The most recent general structural adjustment loan in this data ended in 2009, and the World Bank has since utilized development policy loans (for information on these see http://www1.worldbank.org/publicsector/pe/befa05/OP860.htm and https://ieg.worldbankgroup.org/topic/development-policy-financing-dpf) which include adjustment instruments. In order to examine the robustness of these findings, I also ran the models presented below for the time period ending in 2009. With this limited sample, the coefficients vary in magnitude but the results are consistent with those presented here where in the model including all controls the presence of a SAL does not have a significant association with infant mortality while health projects are associated with lower rates of infant mortality, but only net of controls. Further, I ran the same models after combining structural adjustment with adjustment development policy loans, which yield conclusions that mirror those discussed here.

3 Including country and year fixed effects in the models yield results consistent with those reported here.
